# Understanding the Trajectories of Women who use Violence Through an Intersectional Feminist Analysis

**DOI:** 10.1177/08861099231159653

**Published:** 2023-03-20

**Authors:** Dominique Damant, Carole Boulebsol, Valerie Roy, Matis Trudeau

**Affiliations:** 1 School of Social Work, 5622 University of Montreal, Montreal, Quebec, Canada; 2 Sciences Humaines Appliquées, 5622 Université de Montréal, Montreal, Quebec, Canada; 3 School of Social Work, 4440 Université Laval, Quebec, Canada; 4 School of Social Work, 14846 Université du Québec à Rimouski, Rimouski, Quebec, Canada

**Keywords:** intersectional feminism, trajectories, women's violence

## Abstract

This article discusses the results of a collaborative research project aimed at understanding the life trajectories of women who have self-identified as having used violence in a context other than self-defense, which is an understudied topic. Based on semi-structured interviews and aided by an intersectional feminist framework applied to life course theory, we present a qualitative analysis of 26 women's experiences of violence, precarity, and services. The three groups of trajectories are distinguished by level of precarity as determined by the experience of violence in childhood, socioeconomic and family contexts, criminalization, intensity of violence, and whether the women received adequate support. This shows (1) the need for interventions to prevent the reproduction or aggravation of violence suffered and perpetrated; (2) the importance of considering the inter-related factors (gender, class race, etc.) that contribute to the women's precarity; and (3) that these factors must be considered to understand the contexts in which women have come to use violence, without trivializing or excusing it, but rather properly situating it with a view to better preventing and intervening in these situations. Our recommendations are aimed at ensuring that social work practices do not contribute to the enforcement of punitive measures, but support women in pursuing their path.

In Canada, 25% of those alleged to be responsible for crimes reported to police in 2017 were women, and, of these, 28% were charged with a violent Criminal Code offense (Savage, 2019). According to Pate (2019), racialized women (Indigenous, South Asian, and African Canadian) are the fastest-growing prison population in Canada and “82 percent of women in prison are jailed as a result of behaviour related to attempts to cope with poverty, histories of abuse, and addiction and mental health issues” (p. 1). Violence committed by women has yet to be investigated in depth (Chbat, 2017; Robitaille & Cortini, 2014). To this end, from 2015 to 2018, we conducted a collaborative research project in Québec, Canada, in close partnership with grassroots feminist support and advocacy groups. In accordance with their organizational and political needs, the research analyzes the life stories of 26 women who self-identified as having used violence in a context other than self-defense. Our objectives were to reconstruct the women's life trajectories, understand the imbricated normative expectations at work, describe the women's living conditions, and ascertain the different rationales leading to their use of violence. This article considers their trajectories in terms of their living conditions, experiences of violence suffered and perpetrated, and the services they received. Our study shows (1) the need for interventions to prevent the reproduction or aggravation of violence suffered and perpetrated; (2) the importance of considering the inter-related factors (gender, class race, etc.) that contribute to their precarity; and (3) that these factors must be considered to understand the contexts in which women have come to use violence, without trivializing or excusing it, but rather properly situating it with a view to better preventing and intervening in these situations.

In this article, we review the literature on women's violence. This is followed by the presentation of intersectionality and life course theory as our theoretical framework. Then, we explain our choice to use a qualitative and life story methodology. This is followed by a description and discussion of the results, based on the grouping of all participants into three categories of precarity. In conclusion, we suggest avenues for reflection and action in relation to the problem under study.

## Women's Violence: A Still Misunderstood Phenomenon

Self-defense is not the only reason that women use violence. They may also use it to coerce, pressure, or dominate others. Le Goaziou (2018) offers a critical analysis of theoretical, legal, and social interpretations of violence perpetrated by women. She identifies it as both legally and socially transgressive, noting that normative expectations are that women be docile, empathetic, and to some extent dominated or, at least, submissive. When we accept that women may use violence, we can explore alternative interpretations of their behavior.

There is no consensus on the typical profile of women who use violence (Robitaille & Cortini, 2014). The overwhelming majority of articles we reviewed emerge from the fields of psychiatry and criminology, and few reflect a gender-based analysis. Women who commit violence appear to have a long history of victimization and live in more precarious living conditions than those who do not (Broidy et al., 2018; Dehart, 2018; DeHart & Moran, 2015; Swan et al., 2008; Trauffer & Widom, 2017). They are likely to have health and substance abuse issues, be young, and live in single-parent households (Broidy et al., 2018; Trauffer & Widom, 2017). It is also documented that many have been, or are still experiencing intimate partner violence (DeHart, 2008, 2018). Incarcerated women in particular also have long trajectories of victimization (DeHart et al., 2014; Mackay et al., 2018; Murdoch et al., 2012; Salisbury & Wan Voorhis, 2009). They are more likely to present mental health issues, histories of abuse and substance use, and to have experienced family difficulties and poverty as children (Brennan et al., 2012; Kubiak et al., 2013; DeHart et al., 2014; Estrada & Nilsson, 2012; Rossegger et al., 2009; Salisbury & Van Voorhis, 2009; Taylor et al., 2018). Some of the incarecerated or previously incarecerated women are introduced to criminal activity in their families, including being forced into prostitution or theft (DeHart, 2008).

The strong association between women's victimization and criminalization has been conceptualized as a trajectory from victimization to crime (ibid.). Furthermore, women incarcerated for violent crimes frequently report severe physical abuse over their lifetime. There are 10 times more likely than incarcerated men to have been sexually abused as children (Rossegger et al., 2009; Trauffer & Widom, 2017; Mackay et al., 2018). Following incarceration, many are marginalized and suffer more social exclusion than men (Estrada & Nilsson, 2012).

Most studies of women's violence have adopted quantitative methods. Such research seeks to examine the intertwining of biopsychological and social risk factors for predictive purposes. This strategy stops short of considering women's behaviors based on anything other than the traditional male models: An approach that has been strongly criticized (Dutton et al., 2010). As a result, most of the time women's violence is negated, or on the contrary, deemed a female characteristic subordinate to, or in reaction to male violence (Cardi & Pruvost, 2012). We align ourselves with scholarly critiques of this approach, who point out that the influence of a gendered and racialized social context, for example, is not considered in these studies. Not only does women's violence differ from that of men, but the reactions of people around them (family, school, and state) differ as well, which influences the trajectories.

Qualitative approaches, with a systemic and feminist reading of violence, analyze trajectories to gain a better understanding of violent women without essentializing, trivializing, or psychiatrizing the perpetrators (Alper, 2014; Chesney-Lind & Pasko, 2013; Salisbury & Van Voorhis, 2009). This was the perspective we adopted for the research presented in this article.

## Theoretical Reference Points

In this study, we used an intersectional life course approach to develop our theoretical framework, in concordance with the women's group’s ideology who participated in all the process. The social positions of women and men are not comparable, as they are still shaped by historically unequal gender relations, which may also be modulated by the effects of racism, neocolonialism, and capitalism (Damant & Guay, 2005; Dragiewicz & Dekeseredy, 2012; Johnson, 2011). Research on trajectories that is camped within a feminist analytical framework examines women's violent behaviors by exploring the links between victimization and the use of violence (Alper, 2014; Cardi & Pruvost, 2012; Chesney-Lind & Pasko, 2013; Hamel, 2014; Le Goaziou, 2018; Salisbury & Van Voorhis, 2009; Simonetti, 2016). Such research recognizes that women may resort to violence for reasons other than self-defense, for example, in the case of a mother's violence against her children.

The intersectional paradigm serves as a reminder, among other things, that women are not a homogeneous group. The interconnections of social markers (i.e., gender, ethnicity, class, culture, etc.) must be taken into account as they influence women's trajectories, including political, identity-based, and discriminatory markers (Collins and Bilge, 2016). Richie & Eife (2020)'s publication concerning the experience of Black women and mothers in the child protection and criminal justice systems also informs the data. With an analytic tool called the “Violence Matrix” (Crenshaw 1990), the authors illustrate that “gender violence is understood to be a system of interlocking spheres that mutually create and reinforce each other” (p. 883).

For the life trajectories, we have drawn on Bertaux’ (2010) life course approach. In addition to the subjective evaluation of the event by the subject-actor, the life trajectory depends also on the “social worlds” in which events take place, the events’ intensity, and the repetition of these events throughout the person's life (ibid). There is also a “before” and an “after” the event that modifies the trajectory and provokes a readjustment of actions and representations.

In line with Brotman et al. (2020) and Ferrer et al. (2017), we have incorporated intersectional perspectives to analyze the power relations in play among the different actors, within the cadenced time frames and structural dynamics the narratives evoke. We used an intersectional life course approach because it offers a solid theoretical basis for understanding a set of interlocking oppressions and identities. We took into account individual situations and collective conditions, identification and differentiation based on sociodemographic and cultural factors, and the correlation between domination, power, and agency (Brotman et al., 2020).

## Methodology

We conducted this qualitative research project in partnership with grassroots feminist support and advocacy groups (L'R des centres de femmes du Québec and the Fédération des maisons d'hébergement pour femmes). The project received ethics approval from the University of Montréal and Université Laval. In response to needs identified by our partner groups, the objectives were (1) reconstruct the life trajectories of women who self-identified as having used violence in a context other than self-defense; (2) understand the imbricated normative expectations that they have to deal with; (3) describe the women's living conditions; and (4) ascertain the different rationales leading to their use of violence.

### Data Collection Method

From 2016 to 2017, we recruited 26 participants in five different regions of the province of Québec: Greater Montréal (n = 15), Greater Québec City (n = 6), Laurentides (n = 2), Center of Québec (n = 1), and Montérégie (n = 2). All participants were recruited through the groups collaborating in the research, professional references, and by means of posters displayed in social and community services. They were reached in different settings, such as women's centers (n = 11), organizations working on the issues of substance abuse and prostitution (n = 6), transition houses (n = 4), support groups for young adults (n = 3), and child protection services (n = 2). In order to participate, women had to self-identify as having used violence in a context other than self-defense. They also had to be available to participate in two interviews. At the first contact with the research team, these inclusion criteria were validated.

Throughout the recruitment process, the researchers were committed to creating a sample of women with heterogeneous sociodemographic profiles based on ethnocultural background, age, sexual orientation, and the types and contexts of violence perpetrated. These considerations also guided the recruitment strategies and participant selection.

Each woman took part in two semi-structured interviews of approximately 90 min duration. In keeping with the life story method (Bertaux, 2010), the interviews began with the same opening question: “Please tell me about yourself.” Social markers were validated with the participants as we proceeded to ensure a proper understanding of the narrative thread (i.e., identification regarding gender, racialization, economic situation, etc.). Also, the interviewers were trained to take into account potential power relations with the interviewee so they might reduce the risk of participants feeling uncomfortable, insecure or not deserving of respect.

Themes identified as important following the first interview were addressed during the second interview. Similarly, following the first interview, the principal researcher created a timeline that was validated by participants at the beginning of the second interview. The second interview focused more on participants’ perspectives on the meaning of the violence they had committed and the connections they made to their victimization and their past and current living conditions.

### Data Analysis

Two students transcribed and anonymized all interviews (pseudonyms are used in the article). The data analysis occurred in four phases. First, for each interview, a chronological and diachronic thematic timeline was completed. Then, a thematic tree was constructed based on the units of meaning that emerged from the coding of the interviews with NVivo software. Next, we conducted a vertical analysis of each interview to ascertain the meaning women ascribed to their experiences (Paillé & Mucchielli, 2016). Finally, to complement this, a horizontal analysis highlighted the different cross-cutting thematic segments. We structured our analysis on three main elements: living conditions, experiences of violence suffered and perpetrated, and the services they received.

To ensure accuracy and thoroughness, several strategies were used. These included writing feedback notes following the interviews, holding regular team meetings, using inter-judge agreements, and compiling analytical memos via the NVivo software used for data analysis.

The participants were classified according to four criteria of precarity: (1) the severity and repetition of various forms of violence suffered in their different life contexts (e.g., family or work environment) at all stages of their trajectories; (2) situations of poverty or circumstances leading to it (e.g., low schooling, poverty or extreme poverty linked to the absence of work or insecure employment); (3) reported mental health and substance use issues that the respondents believed interfered with their day-to-day activities; and (4) the severity of the crimes for which they were arrested, as well as repeat arrests, incarcerations, or complaints to youth protection services. While the fourth criterion initially referred to arrests and incarcerations for reasons of violence committed by the participants, it was expanded during the analysis to include involvement with the law for reasons related to the other criteria (e.g., drug dealing, armed robbery, or driving under the influence). Based on these criteria, we established three groups: women with a high level of precarity in their life trajectories (Group 1), moderately high (Group 2), and a lower precarity level (Group 3). Following this classification, further analysis was carried out to try to identify other criteria specific to each group. We present them in detail below.

## Results

*Profile of participants:* The sociodemographic characteristics are detailed in the introduction to the sections that present each group. All respondents had been victims of violence, mostly in childhood, and all of them acknowledged that they had used violence against others. The most frequently reported forms of violence were verbal and physical, while the main targets identified were people with whom they interacted in the school environment (classmates, teachers, and staff), spouse and ex-spouse, and their children or those of their partner or ex-partner. Some participants reported having been physically (n = 13), verbally (n = 8), psychologically (n = 7), or sexually (n = 4) abused by their father or stepfather; physically (n = 14), verbally (n = 13), or psychologically (n = 10) abused by their mother or stepmother; and physically (n = 13), verbally (n = 13), psychologically (n = 17), or sexually abused by their partner/spouse or ex-partner/spouse (n = 6) (Damant et al., 2019).

The vast majority of the women lived in poverty, with few having significant schooling or stable jobs that provided them with enough money to live on. Most of their trajectories showed little or no evidence of an exit from trajectories of violence suffered or perpetrated, poverty/extreme poverty, significant drug addiction, or mental health problems. Few had timely and satisfactory access to services that addressed the causes of their problems and not just the symptoms or consequences.

The women who frequented feminist organizations (n = 18) emphasized the quality of the support provided by workers (welcome, respect, listening, availability, etc.) and the creation of significant ties with other women who frequent these organizations. As indicated below, one of the elements that allowed participants to mitigate the harmful and permanent effects of their trajectories was getting validation of their problems and adequate follow-up. These observations, which we will discuss further, constituted elements of differentiation between the women. To highlight this differentiation, Group 1 is presented in detail to illustrate the integration of the four criteria in the trajectory analysis and Groups 2 and 3 are presented in terms of what distinguished them from Group 1.

### Group 1: Trajectories Marked by Significant Precarity, Lack of Adequate Support, and Some Contact with the law (n = 11)

The 11 women of Group 1 were characterized by the high level of precarity of their trajectory. At the time of the interviews, participants ranged in age from 21 to 55, with an average age of 39. Three were racialized women (Black, women of color, and/or Indigenous). Most were single (n = 8) and had no children (n = 7). More than half identified themselves as bisexual or lesbian. The majority (n = 9) reported having had or still having drug or alcohol problems. Nine were receiving social assistance benefits.

Overall, their trajectories were characterized by especially difficult living conditions from childhood onwards: deep poverty, single motherhood, unstable home, job insecurity, or lack of employment of a parent who in many cases was addicted to drugs and/or alcohol. In addition, several women were racialized and identified as bisexual or lesbian, which made them more vulnerable to marginalization.

As a whole, the lives of Group 1 participants were marked by an accumulation of episodes of victimization, starting in childhood and adolescence. All experienced multiple forms of violence, often severe. For example, one participant described waking up to physical violence as a child: “I must have been around 11. My mother woke me up one morning by punching me in the face. Anyway, my nose was bleeding. I had a split lip.” (Clara, 36). The majority were sexually abused, most of the time by men in their family. Most of them ran away, sometimes several times during adolescence. During this time, they connected with people involved in criminal activities and some began to engage in prostitution.

During their childhood, most of the participants began committing episodes of both physical and psychological abuse against people in their immediate environment like mother or classmates.

Despite the extent and severity of the violence which they either experienced, were exposed to, or committed, most did not receive any support, whether formally or informally. For instance, some women did describe having contact with services during their youth, such as placement or therapy. However, these services were mainly aimed at stopping behaviors deemed negative, such as violence, rather than understanding the causes of that behavior. They felt that professional social workers, psychologists, and psychiatrists were unable to identify and respond to their needs. One participant, for example, described the impact of workers’ attitudes such as judgmental comments: “… when I went into foster care, the only thing the psychologist said to my parents was ‘She has all the makings of a delinquent.’ So, I just did what she said” (Linda, 51).

Most women who were placed in care as a teenager reported dissatisfaction. For example, Irène referred to the trauma she experienced as a result of extended periods of isolation at the youth detention center:

They said it was for my own protection, but I think that spending a week in the dark, in a room with a tiny, covered window, and every two hours, they open it to check whether you’re still alive… I was there a long time and that is a trauma that's still with me today (Irène, 29).

In adulthood, most women maintained links with the “criminal world” and were surrounded by “hard” drug users and dealers. Some spouses also had been judicialized for money laundering or armed robbery. This period was also characterized by regular use of cocaine or heroin. Several women were in prostitution/sex work^1^ for varying lengths of time as ways of supporting themselves.

Episodes of violence including sexual violence continued to occur from different attackers such as spouse, family member, neighbor, co-worker, and johns. They also reported an increase in frequency and severity of the assaults, for example, attempted murder.

All the women were living in deep poverty characterized by no stable employment and income assistance benefits. At the time of the interviews, only one participant worked in a field related to the vocational program completed upon her release from prison.

Their living conditions, coupled with periods marked by a weak and criminalized support network, made it difficult to get out of a “familiar” environment even if it presented a risk for them.

Group 1 respondents had the most contact comparatively with the justice system. Some were arrested as teenagers for shoplifting or running away. As adults, episodes of violence increased in frequency, sometimes in severity, and targeted a larger and more diverse group of people. Arrests were also more frequent, and most were incarcerated for offenses resulting in sentences ranging from a few months to a few years (e.g., arson, assault, and armed robbery).

Most women received one or more mental health diagnoses as adults like depression or borderline personality disorder. Most had a history of self-harming behaviors or attempts to harm themselves, with about half of them beginning this behavior in adolescence.

They also received various services from psychosocial, justice, or other professionals in different settings. Maryse reported receiving more support after becoming a mother and noted it was not focused on her needs but were primarily intended to ensure the child's well-being: “But as soon as I had my daughter, it seemed like they got all organized, because, as I saw it, […]She has to have support because she has a baby who might not do well, not develop the way it should if she doesn’t get help” (Maryse, 55).

Some of the women described feeling deplored by the fact that justice professionals inside and outside of penitentiaries focused on the application of coercive and repressive measures. They complained of interventions centered on risk management, regarding drug use, episodes of violence or suicide attempts, or law enforcement reporting the commission of a crime. They would have preferred support to deal with what they considered their most important problems which they identified as poverty, housing, or victimization.

In terms of coercive measures, several women reported the difficult living conditions in prison and the harsh conditions of probation. As one described: “In the hole, you’re supposed to stay three days max. I was there for five, without a shower… The first night I was there… I was naked on the cement” (Monique, 37).

In addition, mothers in our sample were largely critical about their contact with youth protection services. Indigenous and racialized women identified these services as being fraught with racism, as evidenced by this interaction described during an interview:

They had a negative image and because my mother is an Indian. You know, the whole thing about drunk Indians. I’m stigmatized because I was in youth protection right from the beginning. The social worker was really aggressive. The way she spoke to me. Yes, these appointments, I don’t like none of it. (Célina, 42)

Overall, the services that women in Group 1 received were primarily related to mental health issues or criminal behavior, not their experiences of violence, inadequate schooling, or economic insecurity. As soon as they became mothers, most of the services were focused on the child, labeling them as bad mothers and without consideration for their needs. In what follows, we contrast these examples with our analysis of the remaining two groups in our study.

### Group 2: Trajectories of Precarity Punctuated with Formal and Informal Support (n = 11)

The 11 participants of this group were aged between 33 and 60, with an average age of 49. Three respondents were racialized women. Two-thirds (n = 7) were single, but more of them had children (n = 8). The majority (n = 9) received social assistance benefits and two received a permanent disability pension.

Their life trajectories were also characterized by oppressive living conditions and different forms of victimization during childhood, adolescence, and adulthood. However, as we illustrate below, they differed from Group 1 women in that they committed less serious offenses, had access to services for the violence they experienced as youth, and had the support of one or more relatives.

Their youth was spent in a family environment where many social problems were intertwined, for example, having a parent with mental health issues. Most grew up in a context of poverty or extreme poverty, with single mothers or in a large family. Many had the responsibility of caring for siblings or their own parents. Half of them experienced sexual assaults by men in their family environment. All of them acted violently, sometimes to the point of threatening the lives of members of their close network.

Many of the women spoke of repeated episodes of racism outside their family environment, like in agencies that were supposed to help. Mathilde, for example, spoke of her experience while in care: “They (foster family) beat me. They called me fucking N***. They had a young disabled daughter and I was like her slave, or nanny. It was my job to take her out. I had to make her meals. If she went to bed, I had to go to bed too” (Mathilde, 37).

Despite the various forms of violence experienced, one-third of the participants said that they had not received support from their families to end the violence, that the violence was trivialized, or that they were held responsible for it. Most began committing violence before adulthood. In some situations, the participants were violent toward someone close to them one who was regularly violent toward them, although not in self-defense, as in the case of Christine: “I came home and grabbed my father, got him by the throat. My knee was on his throat. His face turned blue. When the police got there, it took four cops to get me off him because I wouldn’t let go. I wanted to kill him. He abused my sister” (Christine, 57).

In this group, half of the respondents had contact with police, judicial, or psychosocial services during their childhood and adolescence. Most of this contact happened following episodes of abuse, or for reasons that could be interpreted as consequences of their victimization such as attempted suicide or substance abuse. Two participants appreciated these services and the abuse stopped as a result. Four others, however, had a more mixed assessment, citing negative experiences related to ineffective services or professionals’ judgmental attitudes toward them.

As adults, many of the women reported relationships with abusive partners who had been in trouble with the law and/or had substance abuse problems. Victimization continued into adulthood in varying forms. Half of the respondents reported having suffered sexual violence. In nearly all cases, the attacker was male and in half of them, it was the woman's spouse. As a direct consequence of the violence or the influence of their spouses, most started to drink heavily and were diagnosed with mental health problems. Almost half the women had already attempted suicide at the time of the research.

While many had been able to hold jobs in a variety of settings when they reached adulthood their living conditions remained precarious. Indeed, most of the respondents lived in poverty, and some supported themselves by becoming involved in the sex industry. Others quit their jobs, either to care for their children, because of mental health issues, or as a result of serious accidents or injuries related to abuse.

Like the women in Group 1, as adults, all of these women had been physically and emotionally violent to different people, most of them toward one or more children. In the following excerpt, Martine describes with regret an act of physical violence against her daughter:

I pushed her against the wall, just inches away from a nail. You know doorways, how you always hang little things up there. So, two inches away from a nail, I thought if I had thrown her just a little higher, it would have gone into her head, you know? (Martine, 54)

In contrast to the women in Group 1, however, the majority of Group 2 participants received informal support from their environment. Some identified family members, like a grandfather, who, from childhood, encouraged, loved, and reassured them. They also identified people around them in adulthood who they trusted and who could provide them with a safety net. Some of them mentioned shelters during suicidal crises, care for children during hospitalizations, or financial support. Louise said that although communication with her family could be difficult, she knew she could count on them: “They gave me financial, psychological and moral support. […] At a certain point they didn’t know what to do, but they never gave up hope. They’ve always loved me just as I am. I’m lucky, aren’t I?” (Louise, 50).

As adults, all the women had contact with police or psychosocial services for two main reasons. The first of these was being arrested for committing an act of physical violence. These experiences rarely led to incarceration, and if they did, it was for a few days and not for long sentences as in the first group. Respondents mentioned several grounds for their arrests, including assault and death threats. Second, participants used services to cope with mental health issues such as depression, suicidal crises, or drug or alcohol dependence. Half used psychiatric services.

Women's regard for the services they received was mixed. Many emphasized the effectiveness and the bond of trust created with these professionals. At the same time, others, and sometimes the same women, reported feeling judged, and found themselves in power struggles. Still others, like Martine (54), felt the services were not well adapted to their needs: “I think I had 10 appointments with a worker, a psychologist, I think. But, it was really superficial.” Likewise, the few who were mothers experienced questioning of their parenting skills by youth protection services workers. Women who had received services when they were children had the impression that youth protection staff were already negatively biased toward them when they met with them.

In the case of respondents who had participated in feminist community groups, their comments focused on the sense of belonging based on non-judgment and the similarities among group members: Sylvie, for example, described the difference as such: “It wasn’t at all like a meeting with a psychologist, we just let it out. We have a place where we can say how we’re feeling, we’re not judged, and I trust the other women. They’re going through the same thing as me, only the kind of violence is different” (Sylvie, 33).

In sum, the women in this group followed a trajectory that strongly resembled that of the women in Group 1. They were different in that the violence suffered and committed was less severe, they were able to work, and they had informal support from their loved ones. Lastly, although they were critical of the services they received, they did say that some of the services were helpful.

In the following section, we compare the analysis of the two first groups with the third one. Although it is smaller than the others in terms of sample size, it has different trajectories that are worth briefly highlighting.

### Group 3: Trajectories of Precarity with no Criminalization and Marked by Voluntary Access to Support (n = 4)

The four women in Group 3 met two of the four established criteria described in the methodology section. At the time of the interviews, they ranged in age from 29 to 52, with an average age of 49. Two were racialized. Two were single, one was in a couple, and another was married; three of the women had children. Two were employed, another was receiving social assistance benefits; another was looking after her children full time and her husband was the breadwinner. Compared to Groups 1 and 2, they had had less precarious childhood environments, although their trajectories were also marked by poverty. All were victimized in different contexts, but none reported having been sexually abused, which distinguished them from the other two groups. Although they had suffered episodes of physical violence throughout their lives, they were mostly victims of psychological and verbal violence. Although they acknowledged having been violent, especially as adults, none of them had been in trouble with the law. They had voluntarily received services as adults.

Three of the women grew up in a family environment marked by economic poverty, as well as by the alcoholism of a close family member like mother, father, or grandparent. However, unlike the women in the other groups, most said they had a good relationship with one or both of their parents during their childhood. As children, they experienced episodes of violence, mostly at the hands of family members or people in the school environment, but it was less frequent and committed by fewer perpetrators compared with Groups 1 and 2. Most had engaged in verbal violence as children or teenagers, while only one participant reported having used physical violence. These women did not receive services until adulthood, with the exception of one participant who saw a school psychologist as a teen.

As adults, most respondents still lived in poverty. Only one had a paid job, while the others were on income assistance or had no direct income. They continued to be subjected to violence and the perpetrators were still part of their close network. In addition, they acknowledged being verbally abusive as adults, and sometimes physically abusive to their spouse or children. Unlike the other two groups, none reported experiencing a suicidal episode or significant mental health problems. Similarly, these women were not involved with the justice system and received very few services during their trajectory.

To illustrate the link between less difficulties and less service use than in the other groups, we use the explanation of one of the women in Group 3. She said she did not need psychosocial services, because, according to her, her needs were less severe than others’ needs:

Up to now, no, I haven’t needed a psychiatrist or psychologist. So far… I’m doing OK… with my health and mentally. Even with what I’ve had to deal with. I consider myself strong. But sometimes, sure, there are times when it's, how do I say it, well, I’m really down. But I always tell myself there are people who’ve got it worse than me. Because it's true, there are worse cases (Pauline, 52).

More often than not, services were provided at the respondent's request. For example, participants reported having consulted a psychologist in private practice, a family doctor, or having attended a community organization specializing in perinatal care.

Two racialized women are in this group. One of them, an immigrant woman, mentioned that the language barrier had proven to be a barrier in accessing services:

When I got here, I couldn’t speak the language. Even to say, “I have an appointment.” I remember one time, before coming here [community perinatal centre], I went to the hospital [name of hospital] for an appointment, yeah, it was an appointment with the doctor. Back then, I couldn’t speak the language so I just stood in front of the secretary, I didn’t know what to say. It was really, I was really nervous. Finally, I went to the bathroom and cried my eyes out. It was really hard. (Yasmine, 29).

This trajectory was characterized by violence suffered and perpetrated, but to a lesser degree than in the others. Poverty was present, but again to a lesser extent.

## Discussion

Our comparative analysis of the life trajectories of women who use violence confirms this as an important area of study. Having summarized the points of convergence and divergence between the groups, we will now discuss the results from three angles: (1) the intersection of violence and poverty, (2) the intersection of violence and different experiences of marginalization, and (3) the pervasiveness of inadequate services in most trajectories. Before concluding, we will outline recommendations for social work practice and research. Once again, we underscore the fact that although these elements are illustrative, they are not representative of this population.

### Convergences and Divergences Among Women's Trajectories

In addition to the use of violence, commonalities among all trajectories include socioeconomic status, influenced by education level, employment, and income as well as victimization, and the impact of unequal social relations on the level of precarity. Most had lived in poverty-stricken environments from childhood to adulthood. They were subjected to violence at every period, mostly committed by people close to them.

That said, their trajectories differ in several ways. The first is related to the frequency, severity, and diversity of the types of violence to which they were subjected. Women in Group 3 reported experiencing fewer incidents of victimization. Also, women in Groups 1 and 2 appeared to have been more exposed to social problems in their family environment than those in Group 3.

The third factor is the relationship to services. The women in Group 2 received more services as a result of the violence they had suffered, while those in Group 1 received formal follow-up for behaviors that were considered “disturbing” without regard to the violence they themselves had suffered.

The fourth factor relates to physical violence and involvement with the law. The women in Groups 1 and 2 are those who had been in trouble with the law, but unlike Group 2, the women in Group 1 had been incarcerated for offenses deemed more serious, for longer periods of time, and on multiple occasions. In contrast, those in Group 3 had not been involved with the justice system and incidents of violence committed by the women themselves were less frequent and severe than was the case for the others.

Finally, of the women in the sample who had children, only those in Group 1 did not report using violence against them. Furthermore, one last factor regarding poverty is worth mentioning. Although all participants in this study lived in poverty as adults, the reported socioeconomic hardship of women in Groups 1 and 2 was greater.

### Intersection of Violence and Economic Insecurity

The economic insecurity observed among the participants, both during childhood adolescence and adulthood, is consistent with the results of other studies on the subject (Leisring et al., 2003; Pollock et al., 2006). The cumulative effect of the difficulties and episodes of victimization experienced by the participants in childhood seems to have exacerbated their difficulties in adulthood. These difficulties are associated with severe poverty and social poverty, childhood exposure to their parents’ problems, exposure as adults to their spouses’ problems, and violence suffered repeatedly throughout their lives.

The concurrence of their own victimization with the criminalization of their behavior is also documented in the literature (DeHart et al., 2014; Murdoch et al., 2012; Richie & Eife, 2020). This is what DeHart (2008) conceptualizes as the “victimization crime trajectory.” Their victimization was reinforced by the violence experienced in youth and its consequences such as prostitution by parents or relatives, exposure to drugs, substance use, or runaway. It usually continued into adulthood where women were still experiencing one or more forms of victimization, for example, at the hands of johns, spouses, or people with whom they used drugs. Importantly, the point here is not to excuse or trivialize women's behavior, but to contextualize and trace their life trajectories to see how poverty contributes to the appearance and maintenance of violence, particularly in adulthood.

### The Intersection of Violence and Different Experiences of Marginalization

As our study suggests, marginalization related to school, work, and intimate partner violence, has consequences on women's trajectories, potential criminalization, and use of violence. Women who experienced sexual violence during their trajectory were those who had subsequently been involved in environments at greater risk of violence (e.g., the sex industry) and who were more likely to live in criminalized environments. They also appeared to have experienced more violence, which in many cases resulted in greater use of violence, leading to further incarceration. This is consistent with observations by authors such as Rossegger et al. (2009), Trauffer & Widom (2017), and Mackay et al. (2018) on the high rate of childhood sexual victimization reported by incarcerated women. Following incarceration, women were frequently marginalized and suffered social exclusion, a finding consistent with Estrada & Nilsson (2012).

One-third of the respondents were racialized. Many of our respondents alluded to how people in child welfare systems, prisons, and other institutions tended to engage in racial profiling or, more explicitly, to act in a racist manner. Their accounts of relationships with youth protection services, as children or as mothers, were characterized by power dynamics related not only to their gender, but also to their socioeconomic situation, the language they spoke, and the racialization of their lives. As Joseph (2015) underscores, although most spoke French, their accent (Haitian heritage, immigrant women from East Asian countries) marginalized them.

In line with authors such as Richie and Eife (2020), who, influenced by Crenshaw’s (1990) “violence matrix”, illuminate how systemic racism links to other vectors such as gender or class domination, the experiences reported by racialized women in our sample, particularly their treatment by youth protection services, are clearly not isolated events. Rather, they correspond with the reality of thousands of people directly affected by the systemic racism that still permeates many institutions in Canada and the USA.

### Pervasiveness of Inadequate Services

The majority of women in our sample had not received any services related to violence they experienced throughout their lives. Interventions in childhood or adolescence, principally by social workers, tended to target their disruptive or violent behaviors and did not contribute to the reduction or disappearance of violence in adulthood. On the contrary, violent behavior seemed to have increased over time. Also, the absence of services, for instance, through educational institutions, youth protection, or availability of sufficiently paid work, may have led them to support themselves on the margins in various contexts of criminality and precarity.

Furthermore, the mothers’ reported experiences with child protection services were difficult. They described feeling judged and locked in a dynamic of “mother blaming” (Lapierre, 2019). Significantly, those who had had support from family members or received adequate services experienced less precarity in adulthood compared to those who had no support. Given these findings, we now turn to four major recommendations for social work practice and research.

## Recommendations for Social Work Practice and Research

The first specific recommendation concerns services. When women are accessing services, whether voluntarily or involuntarily, the violence they have experienced at different stages of their lives, and the intensity of that violence should be more fully taken into account. Sexual violence profoundly affects the trajectories of victims. Being immersed in particularly violent worlds at a young age led the respondents into spirals of violence, both as victims and perpetrators, once they reached adulthood. For this reason, we believe that screening and prevention of sexual assault and its consequences should become a central component of interventions for girls.

The second recommendation aims to reduce the economic precarity that characterizes the different trajectories of women who have experienced violence. Most of the women we met had lived in severe poverty for most of their lives. The participants, often raised in single-parent families, did not benefit from socioeconomic conditions that would have allowed them to obtain an education or training leading to adequately paid employment. Only one of the respondents, who had access to training upon leaving the penitentiary, was able to change her trajectory. At the same time, those who grew up in a family environment where living conditions were less difficult were also those who presented the fewest elements of precarity as adults. This result supports the development of accessible prevention services to provide economic and psychosocial support to single-parent and vulnerable families. Needs such as a minimum livable wage, equal pay, and childcare for students are struggles that community social workers can take on.

The third recommendation is to recognize and acknowledge the effects of systemic racism. All the racialized women referred to situations of explicit racism or profiling, in addition to the effects of violence and difficult living conditions. Given the cumulative effect of various systems of oppression, our analyses suggest that both practitioners and researchers be reminded to be aware of the intersection of sexism and racism when considering the sources and effects of violence in general and precarity in particular. More specifically, this consciousness encourages practitioners to understand their own biases and privileges and avoid social or racial profiling and discrimination.

The fourth recommendation is that practitioners refrain from trivializing, ignoring, or stigmatizing the violent behaviors of girls and women, but rather take the time to understand their origins, determine the factors of oppression in their environment and consider them as the primary experts on their situation. Also, we retain one of Le Goaziou’s (2018) recommendations, namely “[t]hat it is important to work on singular episodes or cases of recourse to violence, taking care to consider each time the circumstances and contexts, social, economic and political, which can shed light on the passages to the violent act.” (p. 43) [Translated by the authors].

The limitation of the study is the recruitment base, as all participants were frequenting a support organization at the time of the interviews. This means that we did not reach women who did not receive services, although it is possible that their trajectories could be different from those of the participants. Although the results are not generalizable, they offer keys to understanding the trajectories of women who use violence.

## Conclusion

In conclusion, we think that the most important transversal recommendation concerns social work practitioners, teachers, and researchers. Rather than consider women's violent behaviors in isolation, we would benefit from seeing such behavior as to a certain extent a consequence of diverse oppressions.

Few people question their own privileges and social position. Acknowledging our own privileges and social position is the first step for an intersectional practice whether it be in practice, teaching, or research (Hulko, 2009). Hudson and Mehrotra’s (2021) synthesis of recommendations identify micro, mezzo, and macro suggestions that include developing a socio-analysis of clients’ problems, questioning structures of power within their organizations, and creating alliances with social movements that address social issues. We fully adhere to these recommendations. In short, all social work programs should be influenced by an intersectional analysis, which recognizes the contingent and dynamic nature of power relations (Hulko, 2009).

Moreover, since from the outset, participants’ living conditions were marked by great poverty, victimization during childhood, and, for racialized women in particular, experiences of discrimination, we must study the external constraints that weigh on their trajectories. In keeping with both the theoretical and political perspectives of intersectional feminism, it is important to gain a thorough understanding of their life contexts and social position. Beyond individual interventions, collective actions against sexism, poverty or racism remain essential in order to promote better living conditions for all.

Furthermore, it is recommended that social workers seek an in-depth understanding of the contexts in which the violence occurs, giving credence to, and paying attention to the interpretations of those most directly concerned. In prevention, such a stance can help reduce both the risk of victimization and the use of violence.

Lastly, an intersectional feminist perspective that can account for the impact of multiple systems of oppression on women's trajectories is also relevant to both scientific analysis and social work practice with a view to better understanding the contexts and means of violence perpetrated by women.

## Notes

1. We chose to use both terms, prostitution and sex work, in order to be faithful to the various expressions used by the women we met.
